# Toxicity of House Plants to Pet Animals

**DOI:** 10.3390/toxins15050346

**Published:** 2023-05-19

**Authors:** Zuzana Siroka

**Affiliations:** Department of Animal Protection and Welfare and Veterinary Public Health, Faculty of Veterinary Hygiene and Ecology, University of Veterinary Sciences Brno, Palackeho tr. 1946/1, 61242 Brno, Czech Republic; sirokaz@vfu.cz; Tel.: +420-541-562-779

**Keywords:** aroid plants, bulb plants, euphorbias, lily, cyclamen

## Abstract

Cases of ingestion of indoor poisonous plants are relatively common among animals and lead to both acute cases of poisoning and long-term exposure to harmful substances and chronic damage to the animal’s health. Plants produce a large number of secondary metabolites, which serve to protect the plant from attacks by insects, parasitic plants, fungi or, for example, during reproduction. However, these metabolites can be toxic if ingested by animals or humans. Toxicologically effective components found in plants are mainly alkaloids, glycosides, saponins, terpenes and others. This review article describes in detail the most common and popular indoor poisonous plants grown in Europe, the mechanisms of action of their active substances and clinical signs of the respective poisonings. This manuscript is supplemented with rich photographic documentation of these plants not found in similar articles, and also includes a description of the treatment of individual types of poisoning.

## 1. Introduction

In addition to primary, vital metabolites, plants also produce secondary metabolites, which serve to protect the plant from attacks by insects, parasitic plants, fungi or, for example, during reproduction. However, these metabolites can be toxic if ingested by animals or humans.

Cases of ingestion of poisonous plants at home are relatively common among animals [1,2,3] and lead to both acute cases of poisoning and long-term exposure to harmful substances with consequent chronic damage of the animal’s health. Bertero et al. (2020) estimate the inquiries on toxic plants at toxicology information centers to be around 5–10% [3]. Poisonings can occur both when a certain poisonous part of the plant is ingested, and in some cases also when it comes into contact with the skin or mucous membrane, with subsequent irritation or damage of the affected area.

Toxically effective components found in plants are mainly alkaloids, glycosides, saponins, terpenes and others [3,4]. In plants, they are found in different proportions and quantities, which, in addition to the type of plant or cultivar in the case of cultivated plants, also depends on external conditions, such as, e.g., temperature, exposure to light or soil composition [5]. Factors that influence the possibility of poisoning in animals include the resistance/sensitivity of certain animal species (e.g., insufficient glucuronidation in cats), as well as the individuality of animals (health condition, nutrition, genetic predisposition, tendency to eat plants in non-herbivorous species, etc.). The age of the animal (the young tend to be more severely affected due to their curiosity and lack of detoxifying enzymes), boredom (especially in single household pets), changes in the household and new plants introduced are other important factors influencing the risk of plant poisoning [1,3]. The prevailing type of plants involved in poisoning may depend also on their popularity, as there are variable trends among plant growers.

This manuscript describes more than 50 of the best-known indoor poisonous plants which pose a risk of poisoning after ingestion/dermal contact in domestic animals, mainly dogs, cats, and possibly cage birds, rabbits and small rodents. It also includes general recommendations for the treatment of poisoning from these plants. The aim of this article is to educate veterinarians and possibly also the general public on the topic of toxic house plants and consequently possibly decrease the number of such poisoning cases.

## 2. House Plants

### 2.1. Aroid Plants (Araceae)

These plants are among the ones most commonly grown in homes and in recent years have gained more and more popularity due to their low maintenance requirements and minimalist appearance. Each of the genera exists in a huge range of species and color variants which may complicate their exact identification by an unskilled person. On the other hand, as the whole family contains similar active substances, detailed identification of the exact variety is not required for the proper treatment of poisoning following its ingestion.

**Figure 1:**
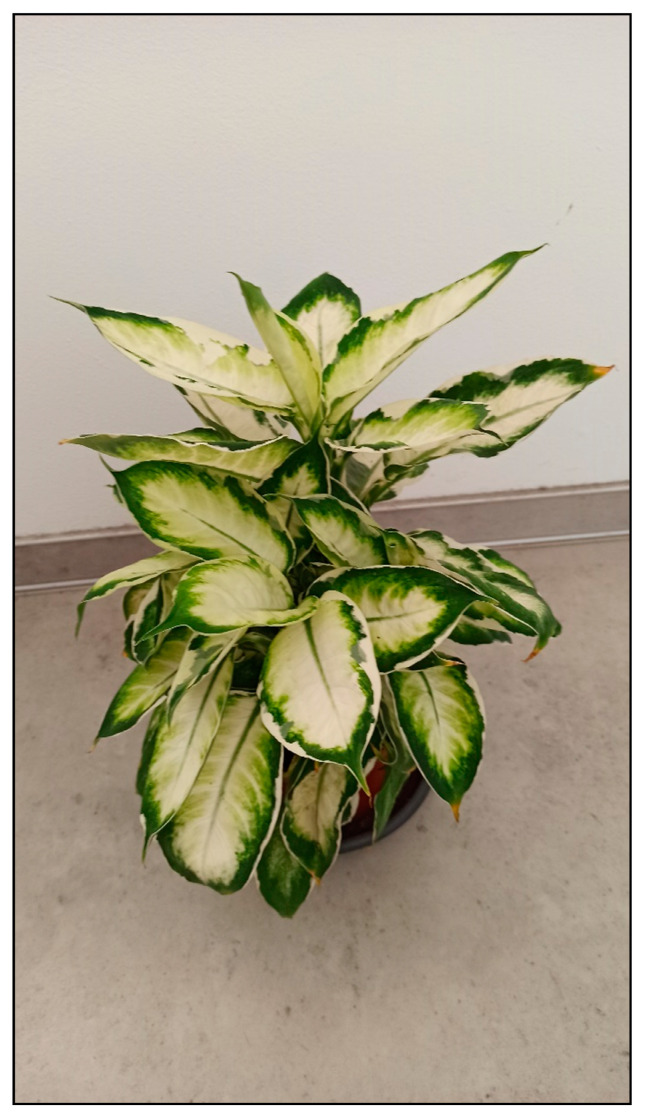
*Dieffenbachia seguine* Cool beauty (contains calcium oxalates; author: Zuzana Siroka).

**Figure 2:**
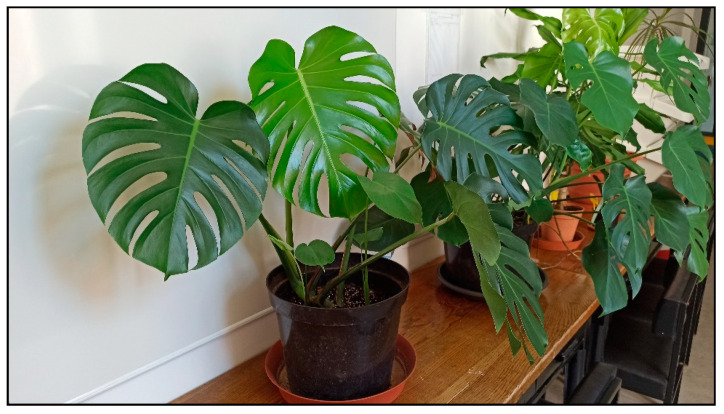
*Monstera deliciosa* (contains calcium oxalates; author: Zuzana Siroka).

**Figure 3:**
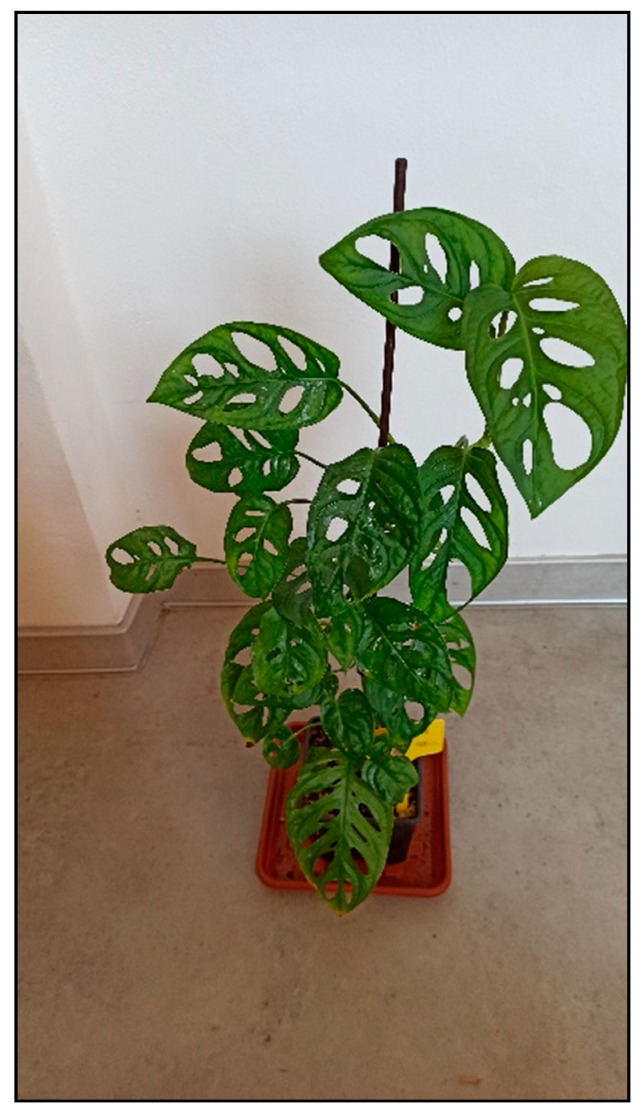
*Monstera aff. adansonii*, monkey mask (contains calcium oxalates; author: Zuzana Siroka).

**Figure 4:**
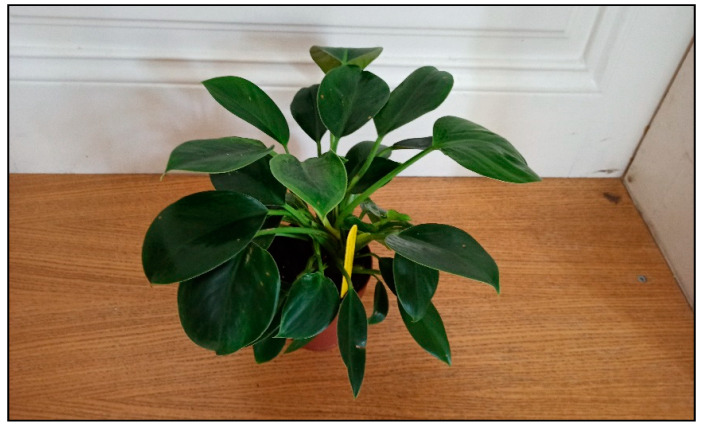
*Philodendron selloum* Green princess (contains calcium oxalates; author: Zuzana Siroka).

**Figure 5:**
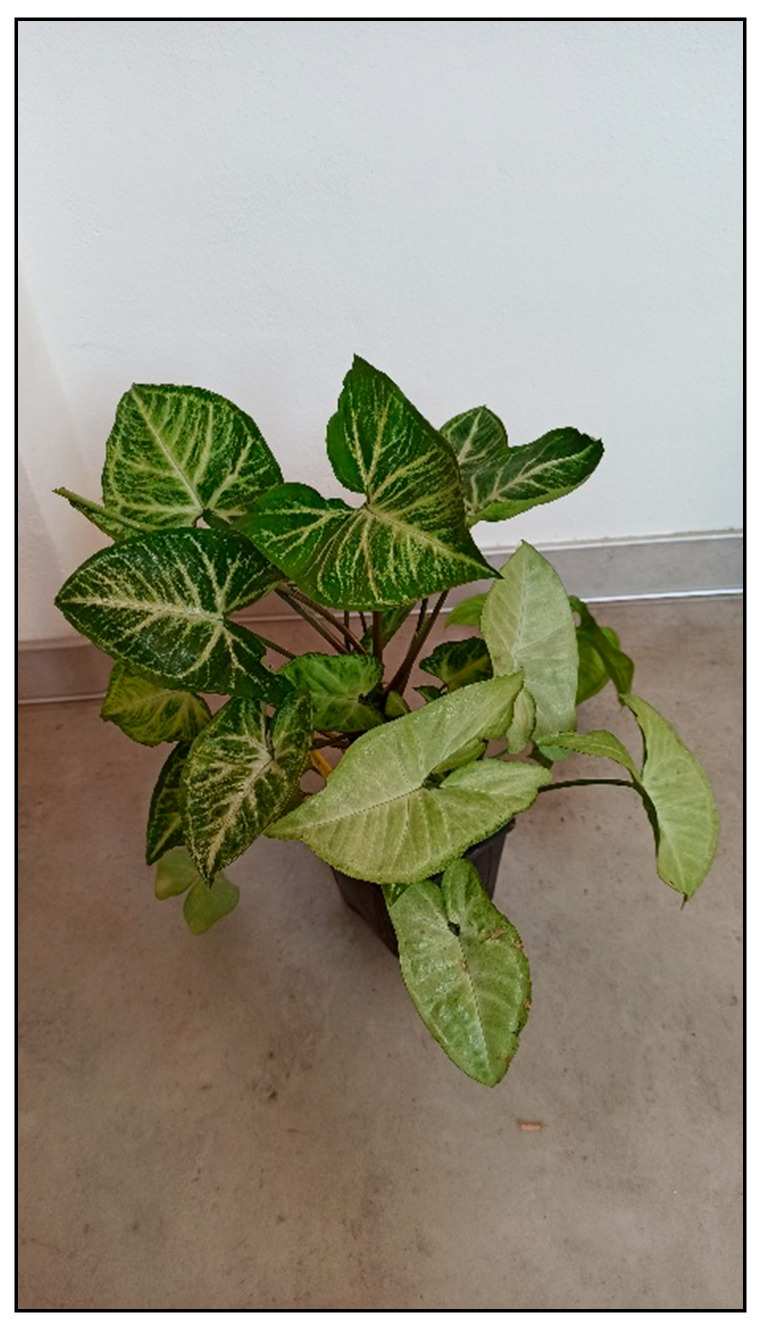
*Syngonium podophyllum* (contains calcium oxalates; author: Zuzana Siroka).

**Figure 6:**
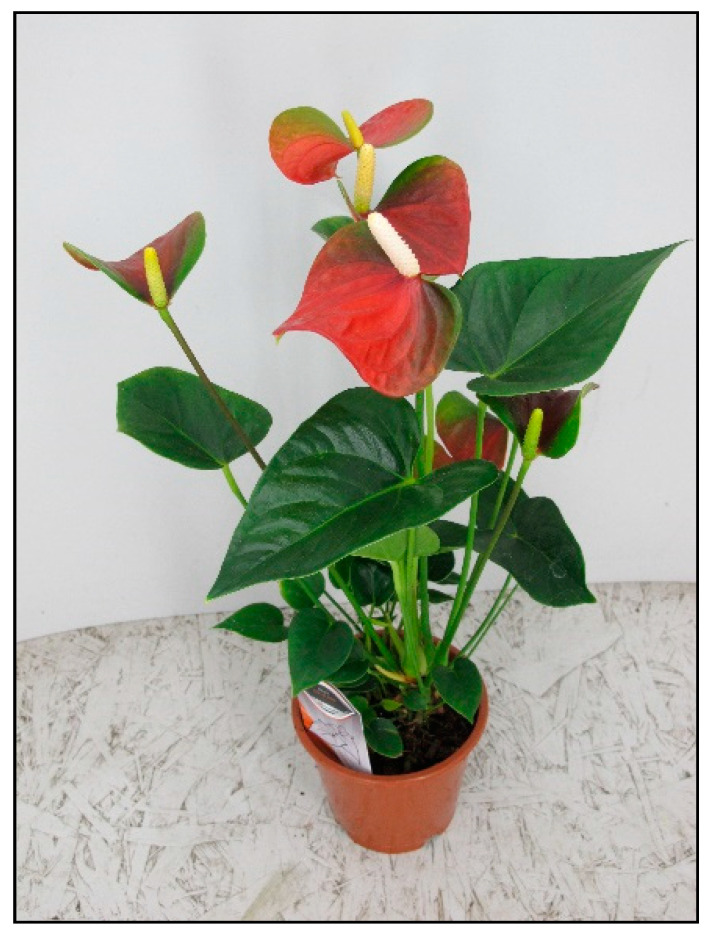
*Anthurium andreanum* (contains calcium oxalates; author: Lenka Divisova).

**Figure 7:**
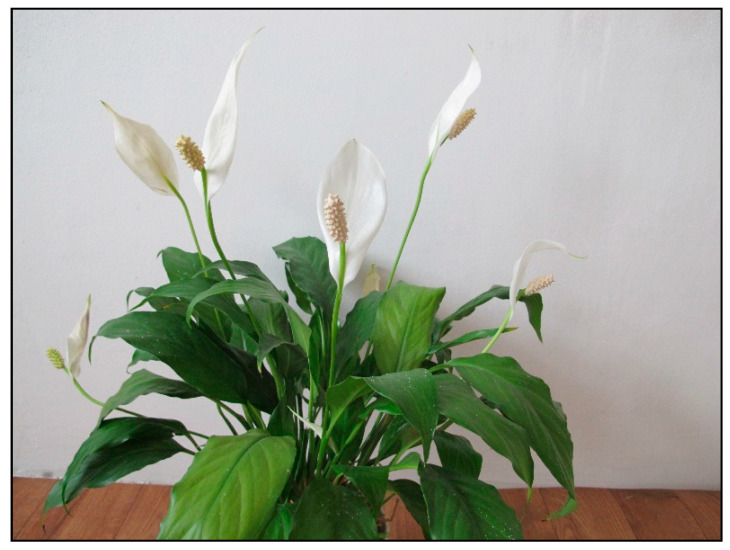
*Spatiphyllum wallissii* (contains calcium oxalates; author: Lenka Divisova).

**Figure 8:**
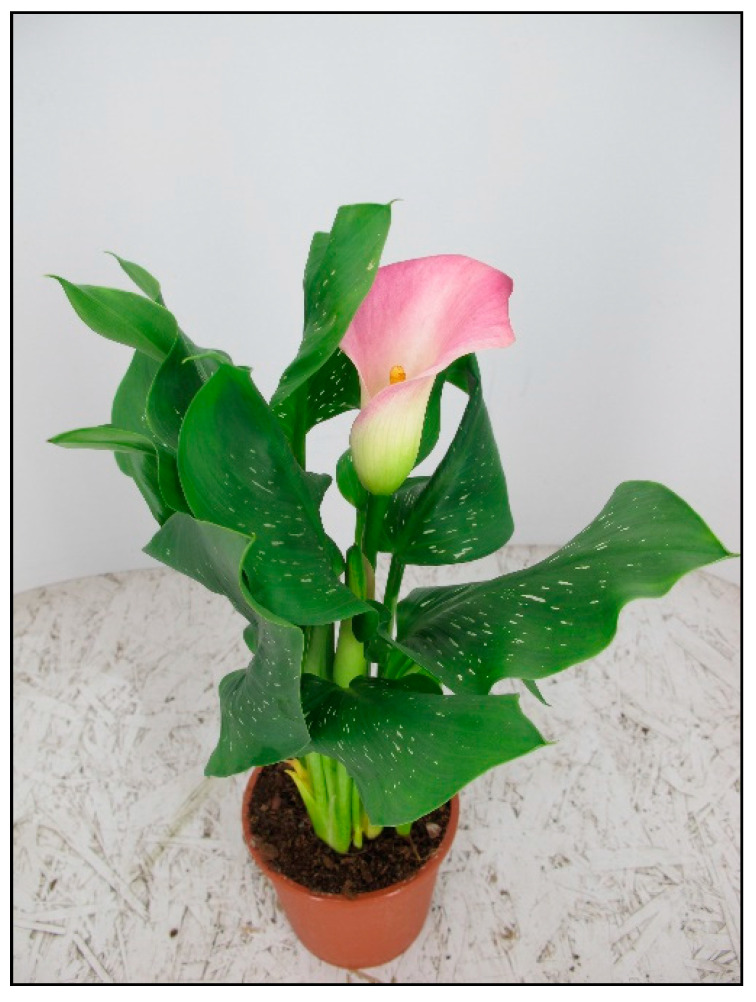
*Zantedeschia aethiopica* (contains calcium oxalates; author: Lenka Divisova).

**Figure 9:**
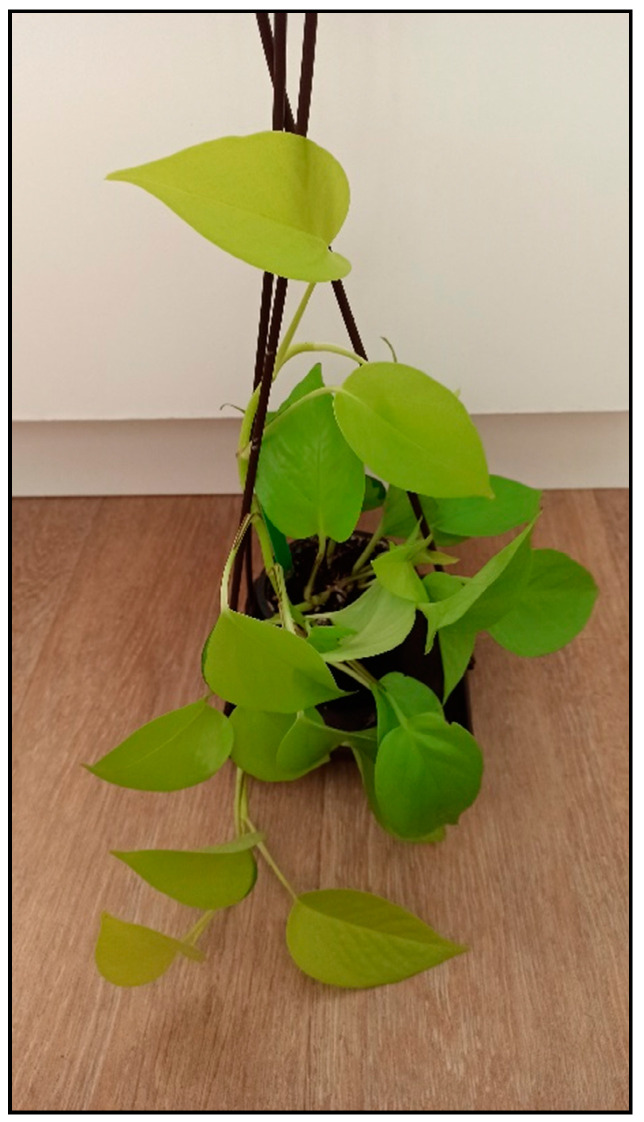
*Epipremnum aureum* Neon (contains calcium oxalates; author: Zuzana Siroka).

**Figure 10:**
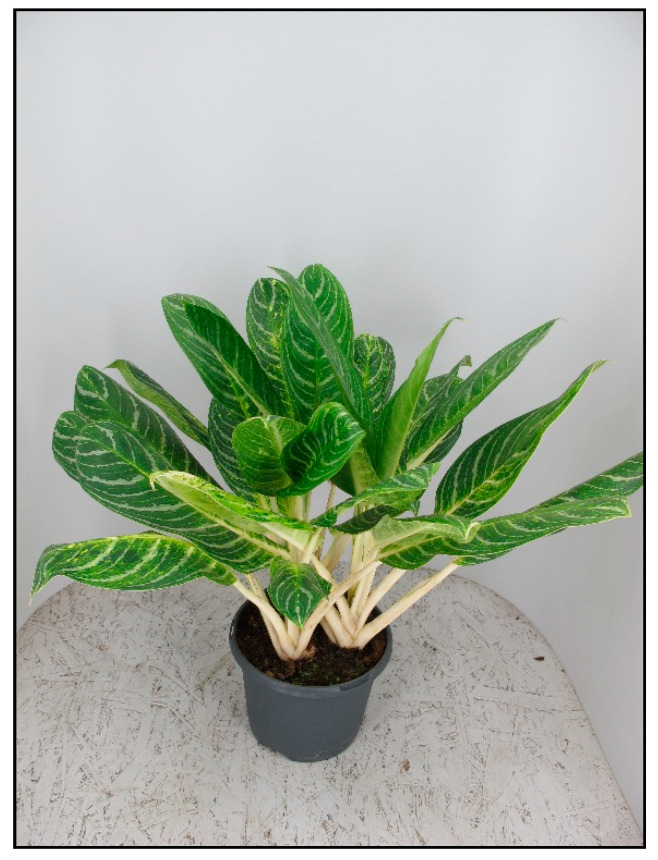
*Aglaonema commutatum* (contains calcium oxalates; author: Lenka Divisova).

Plants from the arum family include, for example, dieffenbachia/dumbcane (*Dieffenbachia* spp., Figure 1), monstera plants (*Monstera deliciosa*, Figure 2, *M. aff. adansonii* or Monkey Mask, Figure 3, *karsteniana*, etc., or *Raphidophora tetrasperma*, known as Monstera minima), philodendrons (*Philodendron* spp., Figure 4), the syngonium/arrowhead plant (*Syngonium* spp., Figure 5), the anthurium/flamingo flower (*Anthurium* spp., Figure 6), peace lily (*Spatiphyllum*, Figure 7), calla lily (*Zantedeschia aethiopica*, Figure 8), devil’s ivy (*Epipremnum* spp., incorrectly called pothos, Figure 9), satin pothos/silver pothos (*Scindapsus* spp.), caladium/elephant ears (*Caladium* spp.), Chinese evergreen (*Aglaonema* spp., Figure 10), alocasia/elephant ears (*Alocasia* spp.), etc.

These plants contain oxalate crystals (oxalates, mainly calcium salts), usually in the form of raphides (needles grooved at both ends) in the ejector cells, as well as oxalic acid and proteolytic enzymes. Oxalates in the form of sharp crystals have irritating effects on the skin and mucous membranes, which, due to their shape, become stuck in the tissues and release histamine from the mast cells, which is then responsible for the symptoms of poisoning. With chronic consumption, oxalates lead to kidney and urinary tract damage. Oxalic acid in excess can cause hypocalcemia. Bromeliad plants, such as tillandsia/air plants (*Tilandsia*), the urn plant (*Aechmea* spp.) and vriesea/the flaming sword plant (*Vriesea*), oxalis/shamrock (*Oxalis* spp.) or begonia tubers (*Begonia tuberhybrida*) have similar effects.

Symptoms include itching, burning, swelling, inflammatory reactions, the formation of blisters on the skin and mucous membranes, hoarseness, salivation, vomiting, and difficulty swallowing. Swelling of the tongue and throat and suffocation are possible. Other symptoms such as severe gastroenteritis with bleeding from the GIT, colic pains, convulsions, and death may also occur. There is a risk of vision damage if raphides become stuck in the eye, with a healing time reaching up to 4 weeks. Philodendron poisonings in cats suffering from nervous symptoms—increased excitability, twitching and convulsions, encephalitis, and temporary (acute) kidney damage/failure—have also been described. Philodendrons, *Epipremnum* and *Scindapsus* have also been identified as allergenic (due to the presence of alkenyl-resorcinol derivates) [5,6,7,8,9,10,11].

### 2.2. Euphorbia/Spurge Plants (Euphorbiaceae)

**Figure 11:**
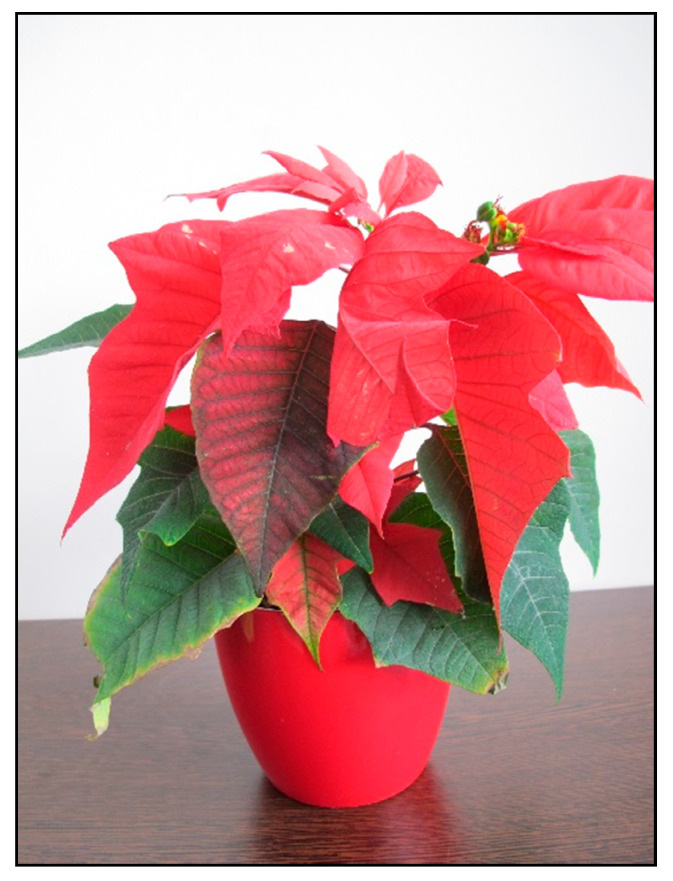
*Euphorbia pulcherrima* (contains diterpens; author: Lenka Divisova).

**Figure 12:**
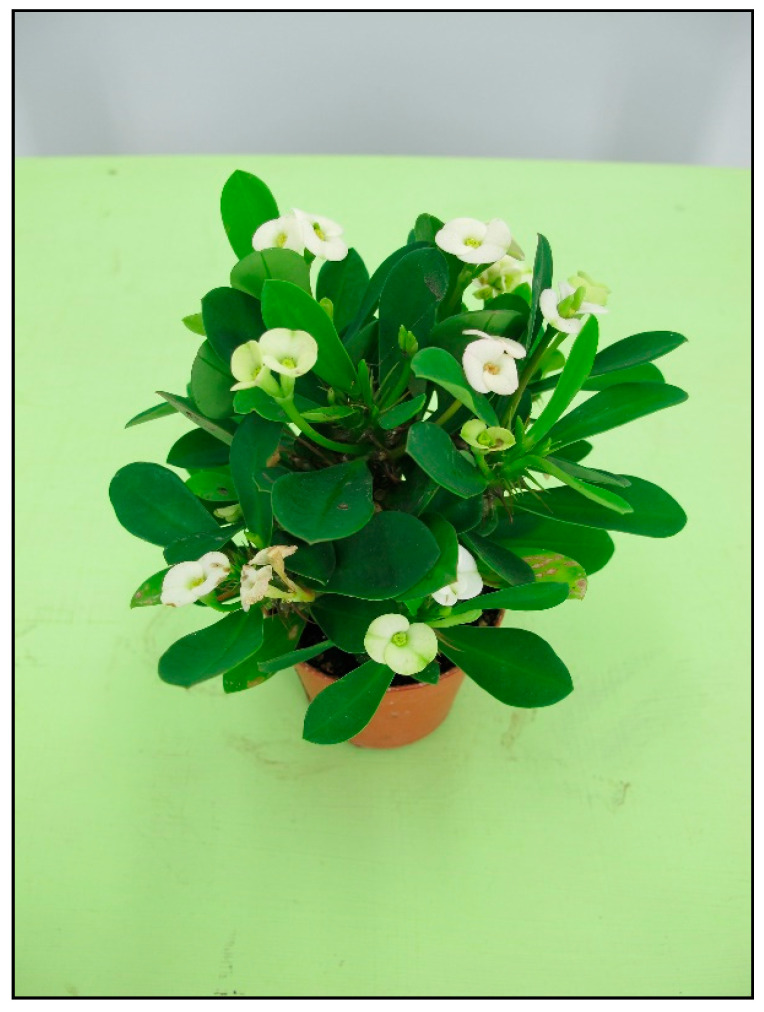
*Euphorbia milii* (contains diterpens; author: Lenka Divisova).

**Figure 13:**
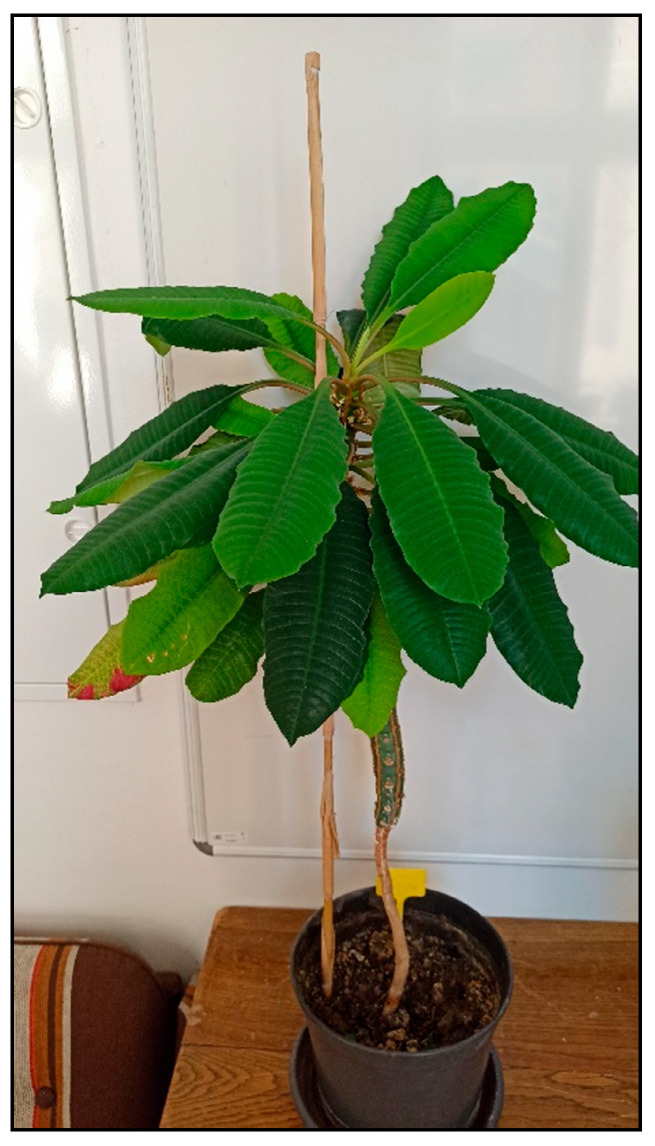
*Euphorbia leuconera* (contains diterpens; author: Zuzana Siroka).

**Figure 14:**
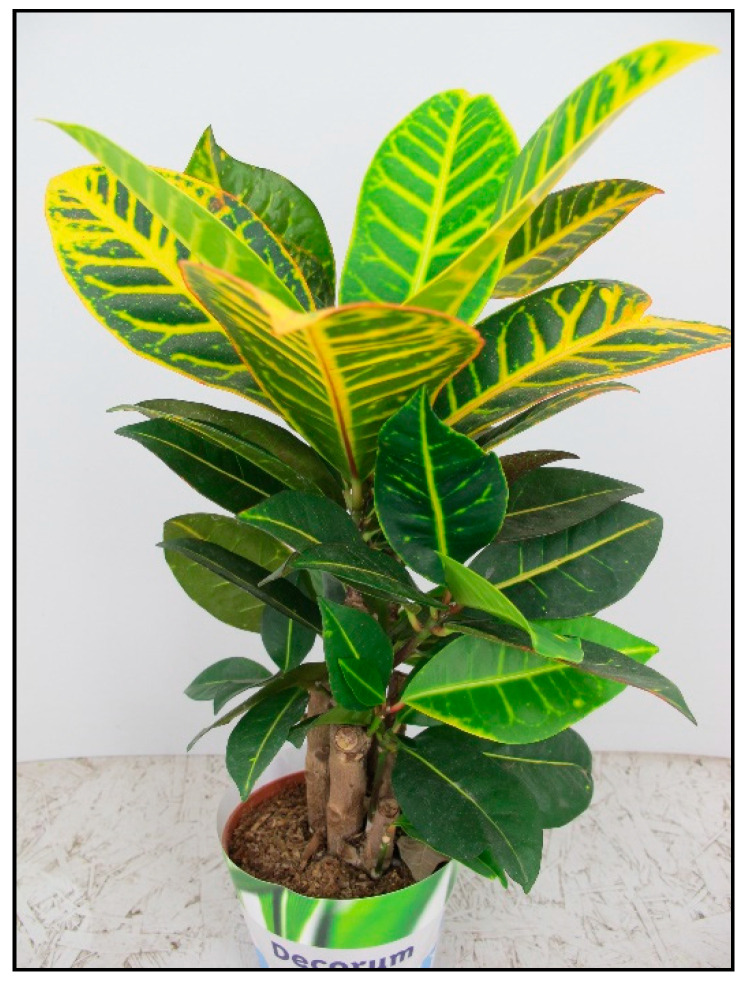
*Codiaeum variegatum* (contains diterpens; author: Lenka Divisova).

Indoor plants from this family include, for example, the poinsettia/Christmas star plant (*Euphorbia pulcherrima*, Figure 11), crown of thorns (*Euphorbia milii*, Figure 12), Madagascar jewel plant (*Euphorbia leuconera*, Figure 13) or croton (*Codiaeum variegatum*, Figure 14). Poisonings are described in dogs, cats, small rodents and birds. Poisonings are usually mild compared to the toxicity caused by outdoor spurges.

After injury, the plants exude latex milk, which contains diterpenes, which are dermatotoxic (causing itching, swelling, rashes, and blisters) and belong to the co-carcinogens (which intensify the carcinogenic effect of other substances and agents).

Contact of the eyes with latex is dangerous, and can lead to swelling of the eyelids, keratoconjunctivitis and corneal erosion. Contact with the skin causes irritation, congestion, rashes, itching, and burning. The local reaction may not be immediate, but symptoms may appear from a few minutes to even several hours after contact with the plant. After ingestion, inflammation of the mucous membranes of the digestive tract occurs with pain, salivation, vomiting and gastroenteritis [5,11,12,13].

### 2.3. Cyclamens (Cyclamen *spp.*)

**Figure 15:**
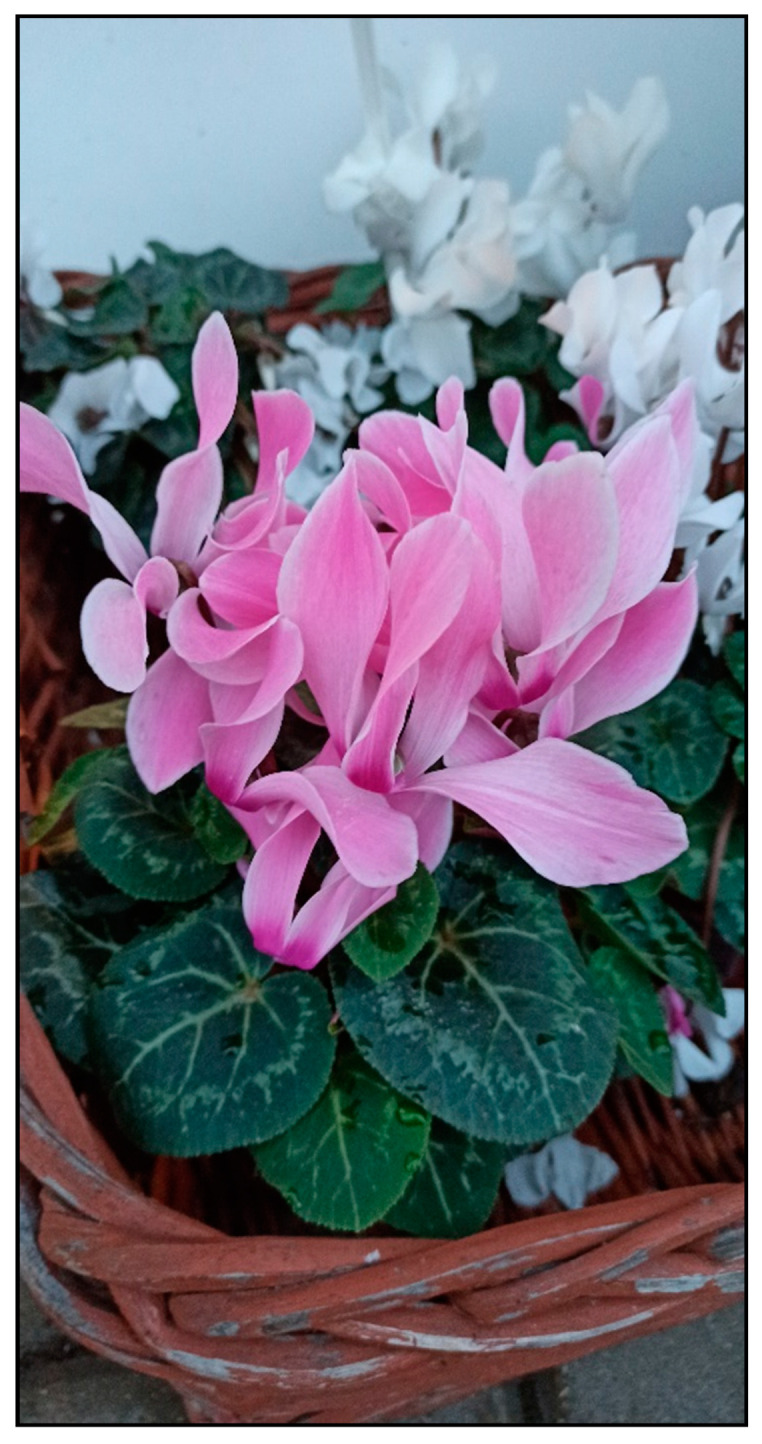
*Cyclamen persicum* (contains saponins; author: Zuzana Siroka).

These plants belong to the Primulaceae family. Persian cyclamen (*Cyclamen persicum*, Figure 15) is mainly grown as a houseplant.

The tubers of these plants contain saponins, e.g., cyclamine, which, unlike most saponins, is well-absorbed from the GIT. Cyclamine has a strong hemolytic effect.

The mechanism of action of saponins is interference with lipids in the cell membrane and a change in its permeability and later integrity. In this way, the cells of the mucous membranes and the skin are affected locally, and after absorption, lysis of erythrocytes also occurs.

Ingestion of cyclamen causes severe local irritation, vomiting, diarrhea, and hemolytic anemia. Occasionally, also convulsions and muscle paralysis, as well as abortions in pregnant animals, occur. As a result of hemolytic anemia, we can observe cyanosis, dyspnea, and hemoglobinuria [5,11].

### 2.4. Dracaena (Dracaena *spp.*), Cordyline (Cordyline *spp.*), Snake Plant/Mother-In-Law’s Tongue (Sansevieria *spp.*)

**Figure 16:**
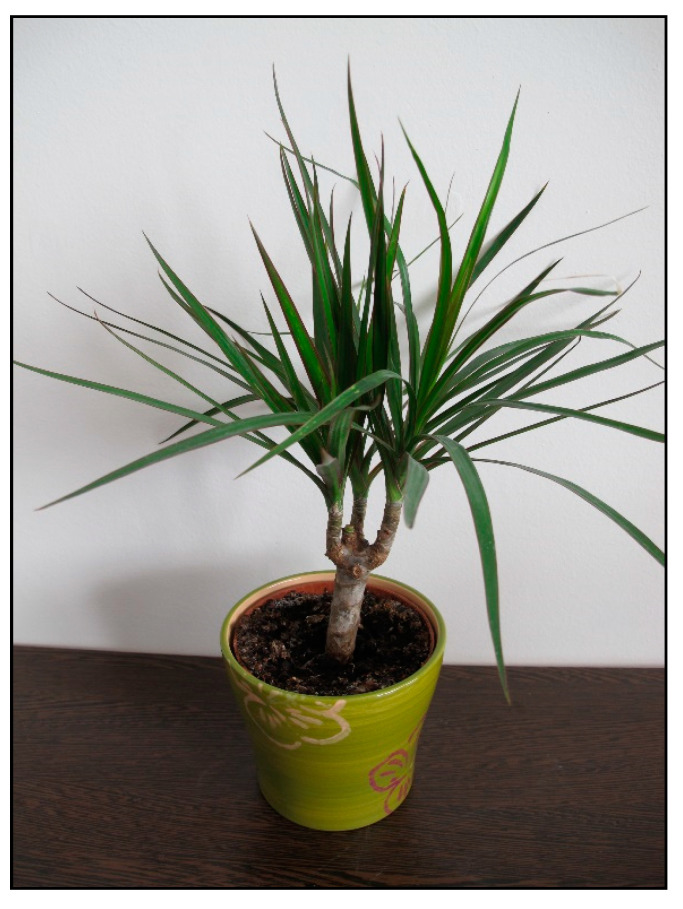
*Dracaena marginata* (contains saponins; author: Lenka Divisova).

**Figure 17:**
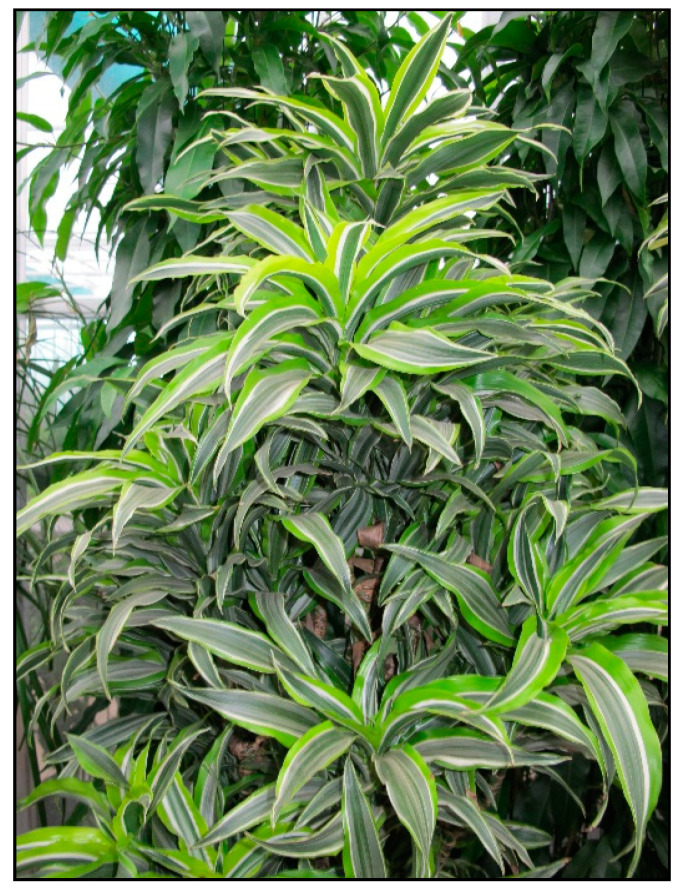
*Dracaena fragrans* (contains saponins; author: Lenka Divisova).

**Figure 18:**
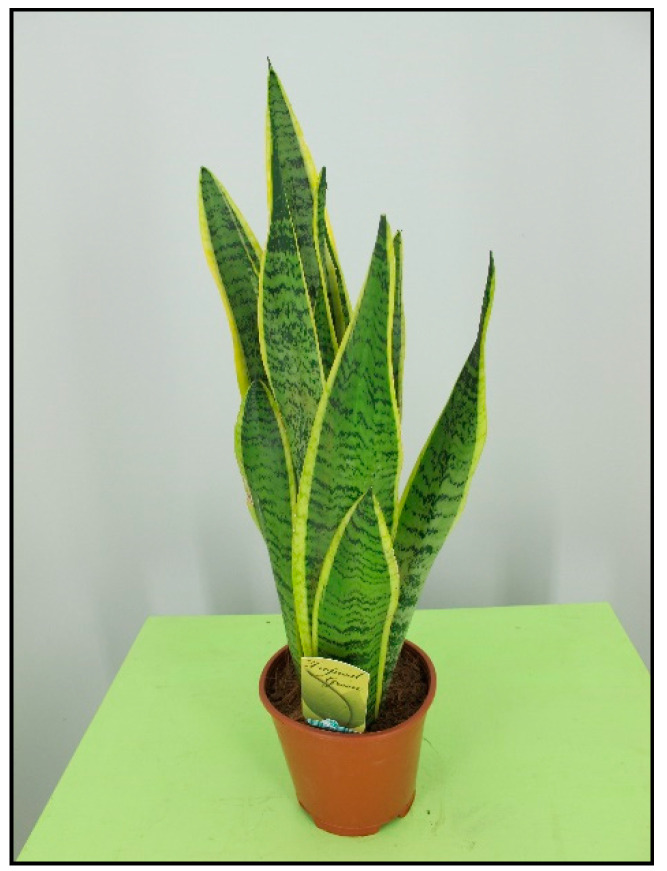
*Sansevieria trifasciata* (contains saponins; author: Lenka Divisova).

**Figure 19:**
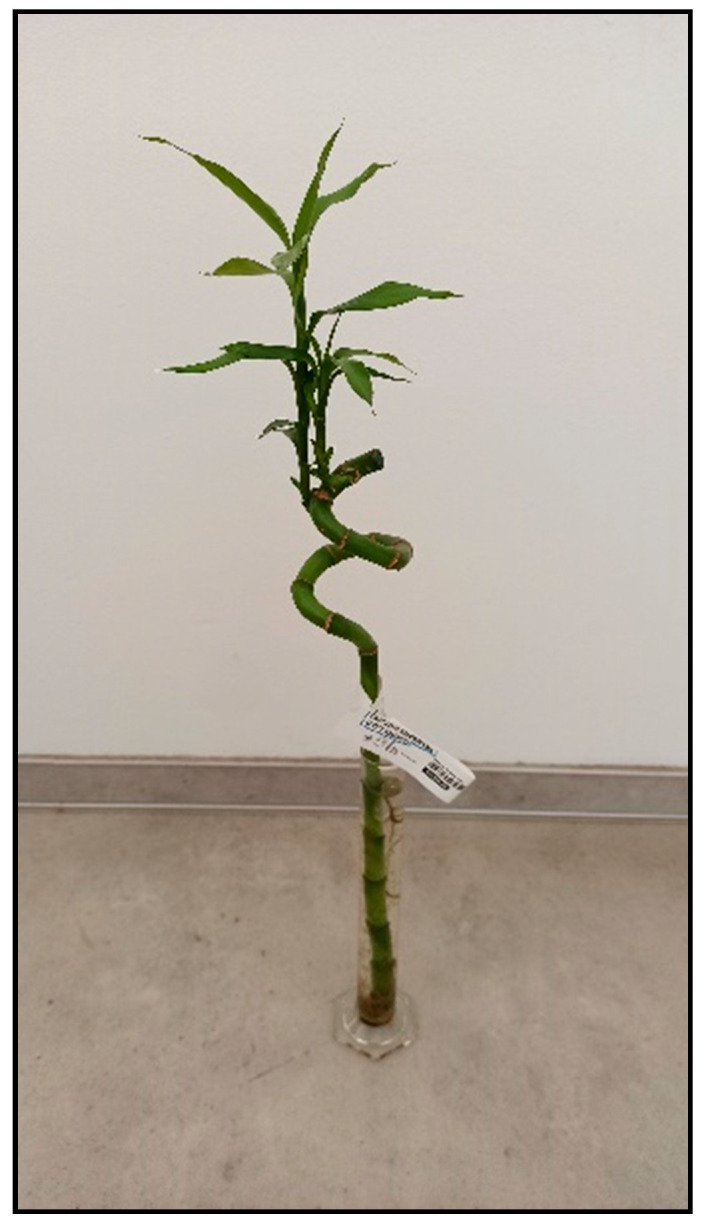
*Dracaena sanderiana* Lucky bamboo (contains saponins; author: Zuzana Siroka).

There are many species and color varieties of dracaena plant (Figure 16 and Figure 17) and cordyline plant. Like mother-in-law’s tongues (Figure 18), they belong to the asparagus family (Asparagaceae), but sometimes they are also included in the agave family (Agavaceae); these families are intermingled. One of the species belonging to this group is *Dracaena sanderiana* (Figure 19), which is sold under the misleading name lucky bamboo.

All these plants contain saponins with an irritating and potentially hemolytic effect. The mechanism of action of saponins is, as mentioned above (see cyclamens), interference with lipids in the cell membrane and a change in its permeability and later integrity.

Ingestion causes local irritation, salivation, vomiting, diarrhea, loss of appetite, depression, weakness and ataxia. Mydriasis and tachycardia are also possible symptoms observed in cats [14,15].

### 2.5. Bulb Plants

**Figure 20:**
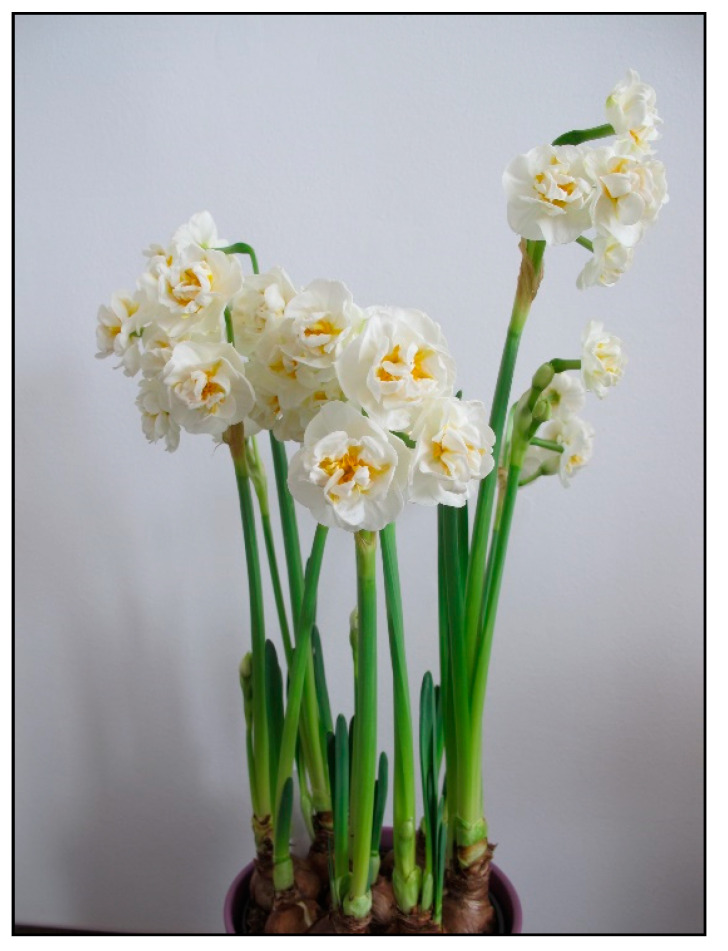
*Narcissus pseudonarcissus* (contains neurotoxic alkaloids; author: Lenka Divisova).

**Figure 21:**
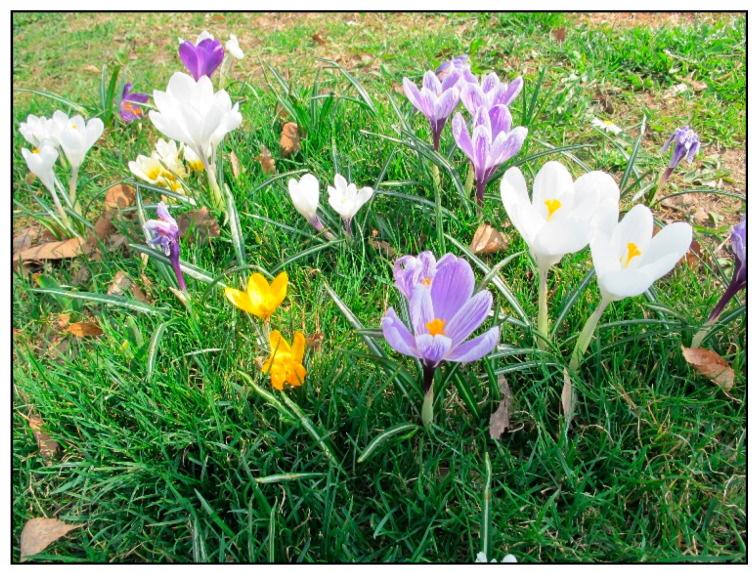
*Crocus vernus* (contains alkaloids; author: Zuzana Siroka).

**Figure 22:**
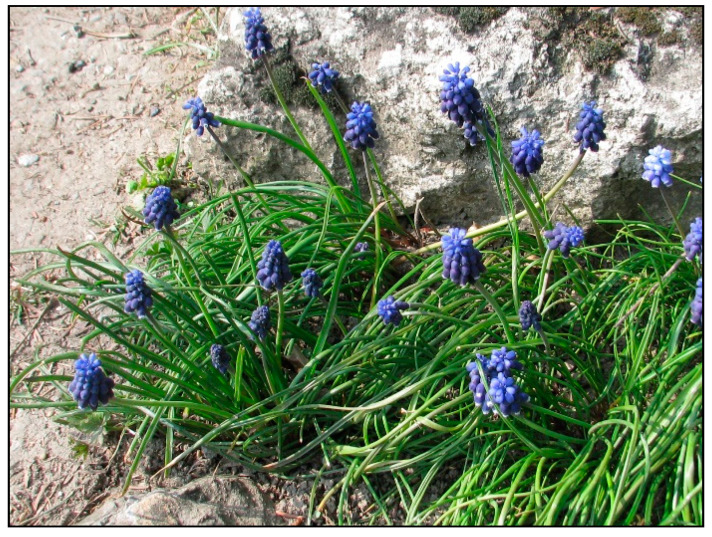
*Muscari armeniacum* (contains alkaloids; author: Zuzana Siroka).

**Figure 23:**
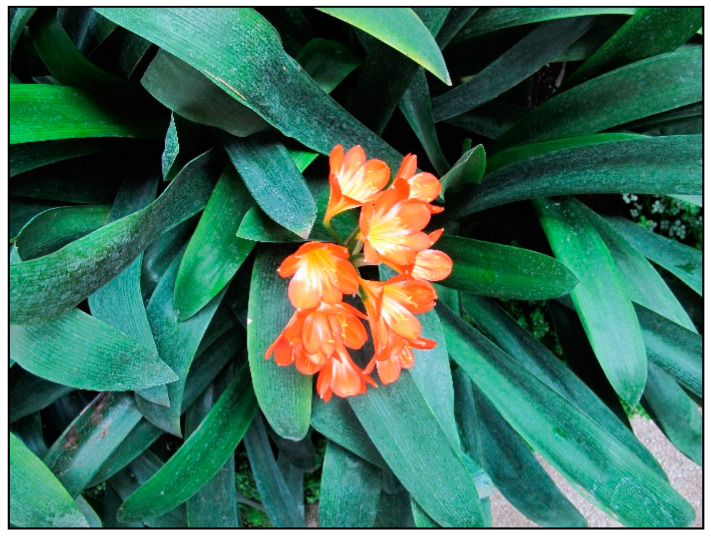
*Clivia minata* (contains alkaloids; author: Lenka Divisova).

**Figure 24:**
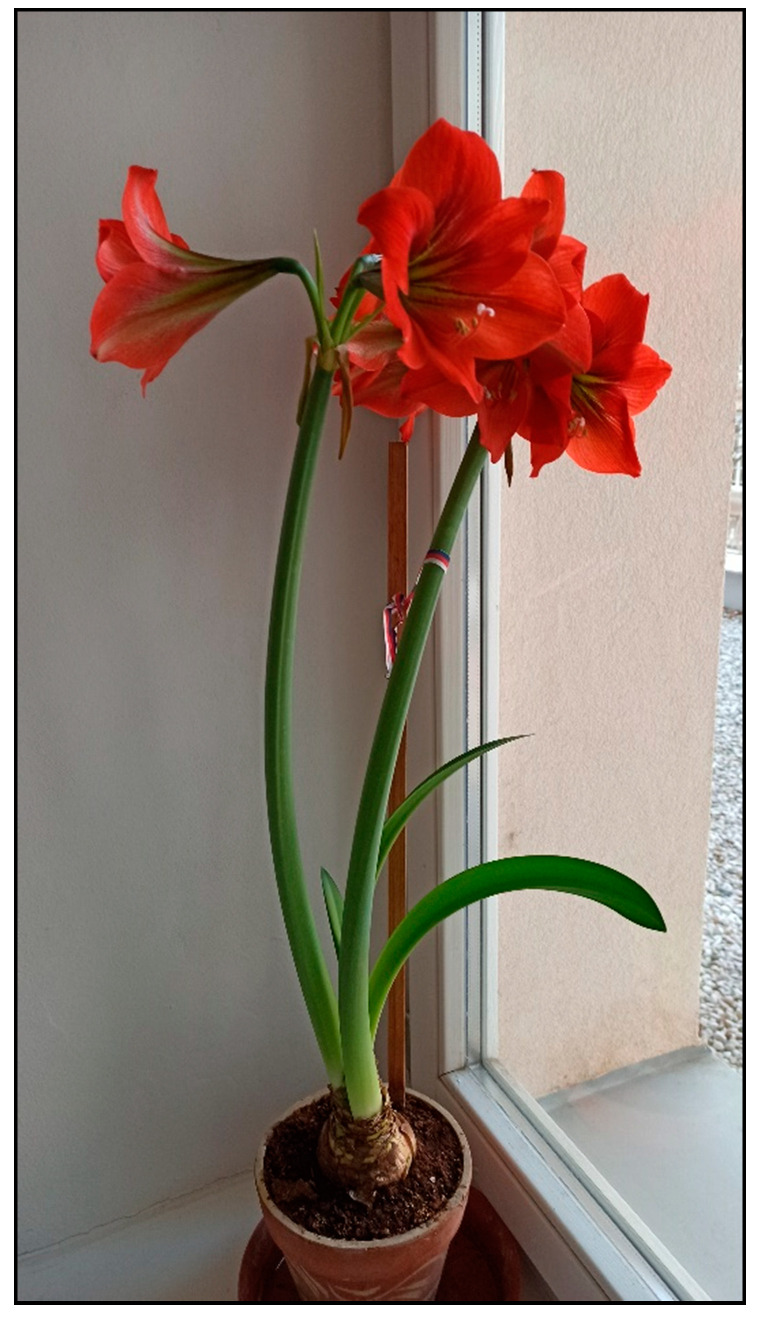
*Hippeastrum x hortorum* (contains alkaloids; author: Zuzana Siroka).

In households, bulb plants appear most often during winter and early spring as single-use decorative plants (e.g., tulips, daffodils, hyacinths, crocuses, and grape hyacinths; Figure 20, Figure 21 and Figure 22), or year-round plants, e.g., clivias (*Clivia minata*, Figure 23), hippeastrum/Barbados lily (*Hippeastrum x hortorum*, Figure 24) or amaryllis (*Amaryllis belladonna*). These plants usually belong to the Amaryllidaceae or Liliaceae families (formerly a joint family).

These plants contain alkaloids, e.g., lycorine, galantamine, tazetine, and oxalate crystals, usually in the form of raphides. The highest concentrations of the active ingredients are usually in the bulb. The exact mechanism of action of substances in most of these plants is not precisely known, but alkaloids are often neurotoxic and cytotoxic. The effect of galantamine, which acts as a reversible inhibitor of acetylcholinesterase (acetylcholine accumulates in the synapse), has been studied. Lycorine also inhibits cholinesterases, inhibits proteosynthesis and further induces apoptosis. Tulips mainly contain locally acting glycosidic substances called tuliposides, which cause allergic reactions and mild to severe contact dermatitis.

Symptoms of poisoning include salivation, nausea, vomiting, diarrhea, arrhythmias (more often bradycardia), and muscle activation or paralysis, coma, and death. Skin reactions or erosions on the mucous membrane of the oral cavity are also common [5,11,16,17,18].

### 2.6. Lilies (Easter Lily, Asiatic, Oriental and Other Lily Hybrids, generally Lilium *spp.*)

**Figure 25:**
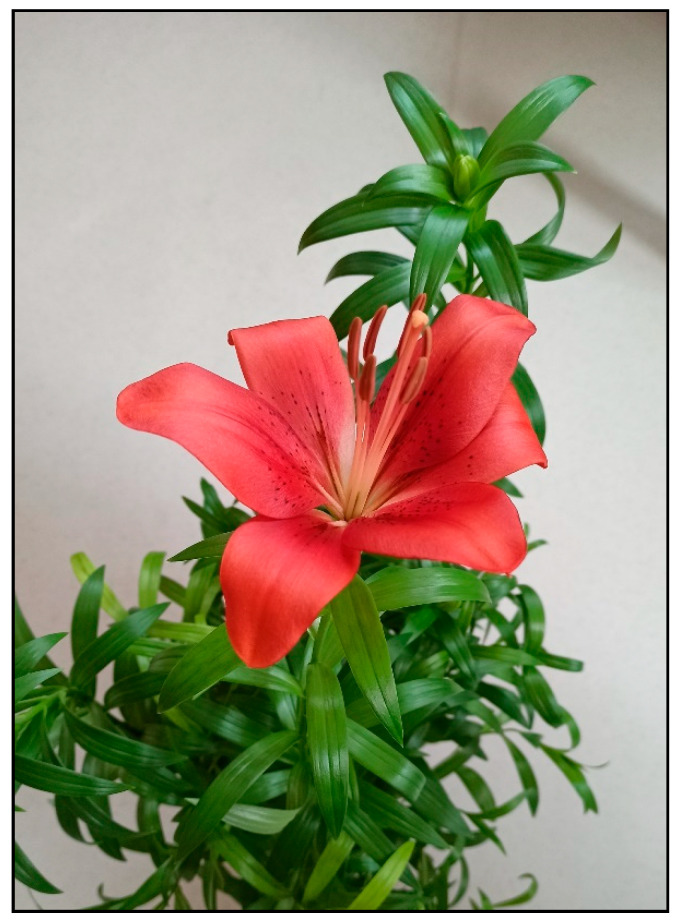
*Lilium* hybrid (toxic substance is unknown; author Zuzana Siroka).

Lilies (Figure 25) are bulb plants that belong to the Liliaceae family; in addition to garden growing, they can be also kept as potted indoor plants and often appear in homes as cut flowers. They are intentionally listed separately in this manuscript, as their type of action differs from that of the other bulb plants listed above. Although they can also cause mild gastrointestinal symptoms due to oxalates, the main risk resulting from consumption lies in the possibility of developing kidney damage or even kidney failure, which is described specifically in cats. The substance that causes renal toxicity is unknown.

It is assumed that poisoning in cats is caused by the lack of some metabolizing enzymes, so in cats an alternative metabolic pathway possibly results in the formation of a toxic metabolite, which does not occur in other animal species. The entire plant, including the pollen, is poisonous, with the lethal dose for a cat being approximately two leaves or petals of the plant.

Poisoning occurs quickly with symptoms appearing within 1–3 h after ingestion. They include salivation, vomiting, loss of appetite, depression, and exceptionally, convulsions after high doses of the plant. Approximately 12 h after ingestion, thirst and polyuria can also be observed. Later (18 h after ingestion), dehydration is evident, and after about 24 h, oliguric to anuric renal failure with vomiting and convulsions due to uremia sets in. A significant diagnostic finding is the disproportionate increase in plasma creatinine compared to urea (BUN) levels, which increase less and more slowly [5,19,20,21].

### 2.7. Common/English Ivy (Hedera helix)

Ivy is grown in many color and size variations both as an indoor and outdoor plant. It belongs to the Araliaceae family and contains triterpenoid saponins (e.g., hedera saponin C) in the whole plant, polyins (also called poly-ynes) in the leaves, and possibly also oxalic acid.

**Figure 26:**
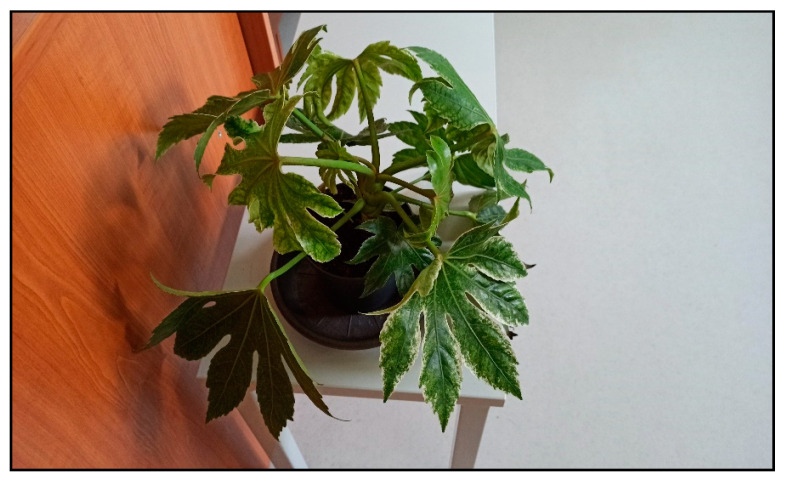
*Fatsia japonica* (contains saponins; author: Zuzana Siroka).

**Figure 27:**
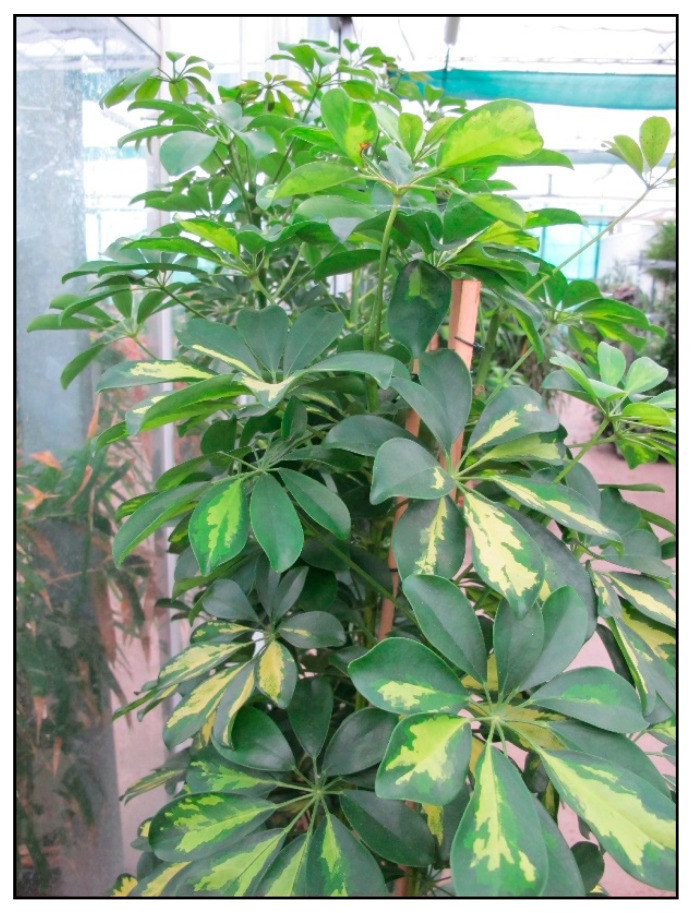
*Schefflera arboricola* (contains saponins; author: Lenka Divisova).

The mechanism of action of saponins is interference with lipids in the cell membrane and a change in its permeability and later integrity. The cells of the mucous membranes and the skin are affected locally, and after absorption hemolysis also occurs. In ivy, damage to hepatocytes is also possible, but usually does not occur in acute poisonings. Polyins (mainly falcarinol and its derivates) are irritants and allergens which interact with proteins in the cells. These are also found in other indoor plants from the Araliaceae family such as *Fatsia japonica* (Figure 26), ivy tree (*Fatshedera*), *Schefflera arboricola* or *actinophylla* (Figure 27).

Saponins cause burning in the mouth, salivation, vomiting, diarrhea, irritation and cough. Fatigue and dyspnea may sometimes occur. Polyins contained in the leaves cause frequent contact dermatitis and allergies [11,22].

### 2.8. Mistletoe (Viscum album)

During Christmas time, poisonings from white mistletoe used for decorative purposes are encountered. This semi-parasitic plant belongs to the Loranthaceae family and contains alkaline proteins, polypeptidic viscotoxins and lectins, which are also proteinaceous in nature. Lectins bind to ribosomes and thereby inhibit them which results in the inhibition of proteosynthesis. Lectins also damage the cell membrane and have a hemagglutination effect. Viscotoxins damage DNA synthesis, affect the permeability of cell membranes and are cytotoxic and hepatotoxic. Since mistletoe is a semi-parasitic plant, its toxicity can also depend on the species of tree it grows on.

Mistletoe causes local irritation of the tissues all the way to necrosis, vomiting, and diarrhea. Further, increased temperatures and flu-like symptoms—fatigue, skin rashes, and allergic reactions—but also changes in heart rhythm or convulsions may occur. Chronic intake damages the liver and lowers blood pressure [5,11,23].

### 2.9. Oleander/Nerium (Nerium oleander)

**Figure 28:**
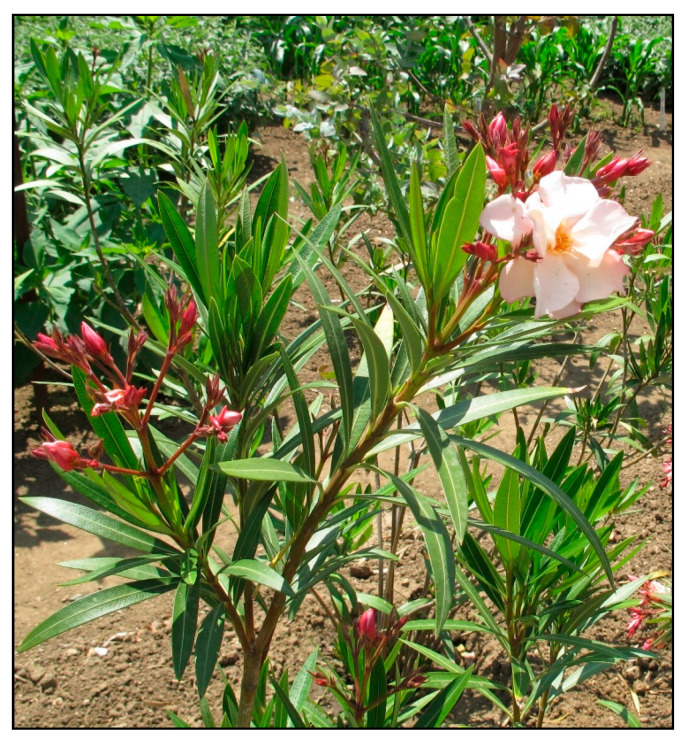
*Nerium oleander* (contains cardiac glucosides; author: Zuzana Siroka).

**Figure 29:**
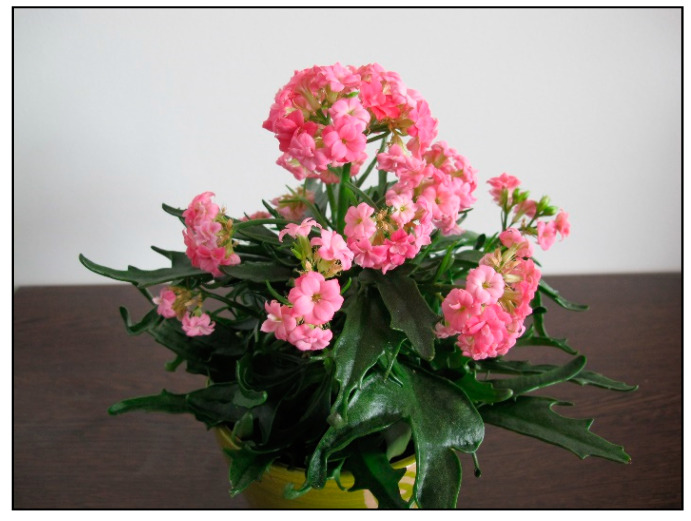
*Kalanchoe blossfeldiana* (contains cardiac glucosides; author: Lenka Divisova).

**Figure 30:**
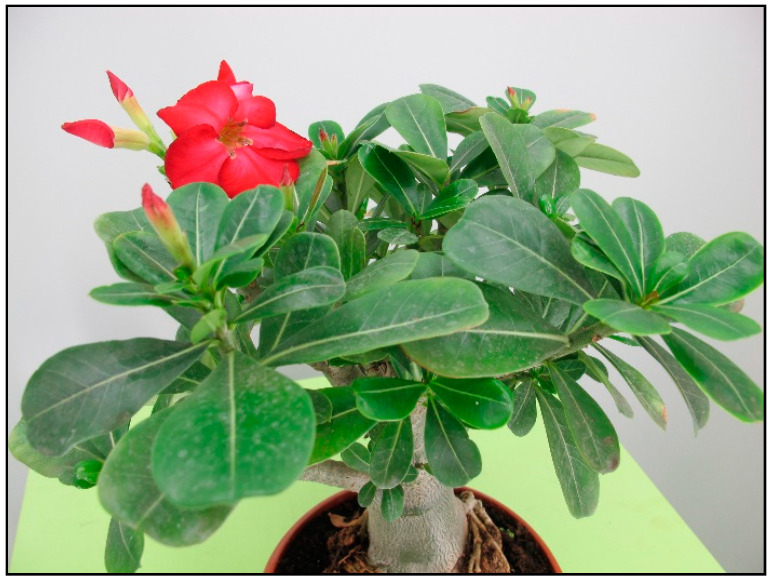
*Adenium obesum* (contains cardiac glycosides; author: Lenka Divisova).

Among other plants causing winter poisoning in domestic animals and summer poisoning in rodents or tortoises kept in summer enclosures is oleander (Figure 28), which must be kept indoors during the winter in many mild- to cold-climate countries, because it is not frost-resistant. It belongs to the dogbane family (Apocynaceae) and contains cardiac glycosides. These are also found in indoor plants such as kalanchoe (*Kalanchoe blossfeldiana*, Figure 29) or desert rose (*Adenium obesum*, Figure 30).

The bitter taste of most cardiac glycosides often does not have a sufficient deterrent effect, but sometimes their ingestion leads to protracted vomiting and thus the absorbed dose is reduced. Cardiac glycosides undergo enterohepatic circulation.

The mechanism of their action is the inhibition of the myocardial form of the Na^+^/K^+^-ATPase enzyme, resulting in sodium accumulation inside the cell, an increase in the intracellular Ca^2+^ concentration, and increased myocardial contractility. Arrhythmias, extrasystoles and fibrillations occur.

Other symptoms include nausea, burning in the mouth, salivation, vomiting, colic, bradyarrhythmia, dyspnea, ataxia, dizziness, hemiplegia, and mydriasis, while visual disturbances and hallucinations may also occur. Extrasystoles, atrial flutter, AV block, ventricular fibrillation, cardiogenic shock, unconsciousness and death occur at high doses. Hypoglycemia and azotemia can occur in dogs with oleander poisoning [5,11,24,25,26].

### 2.10. Plants Containing Pyrrolizidine Alkaloids

**Figure 31:**
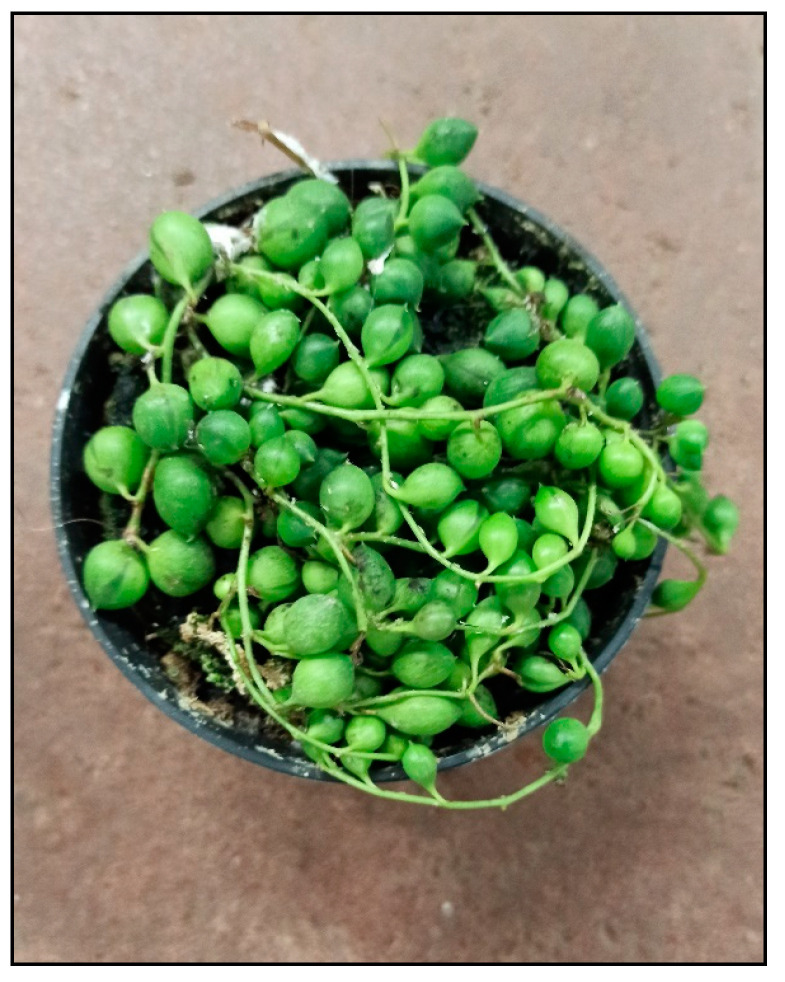
*Senecio rowleyanus* (contains pyrrolizidine alkaloids; author: Zuzana Siroka).

Among the indoor plants, these substances are mainly contained in the genus *Senecio* (e.g., string of pearls, *S. rowleyanus*, Figure 31, or *S. barbertonicus*) from the Asteraceae family. Rabbits and rodents (with the exception of the rat) are relatively less sensitive to these plants. Poisoning usually manifests itself only after chronic consumption of such plants, while acute poisonings are rare. Poisonings from house senecios are less frequent and less serious than outdoor senecio/ragwort poisonings.

In the liver, pyrrolizidine alkaloids are metabolized by cytochrome P450, which bioactivates them by forming reactive pyrroles from them. These then act as alkylating agents and damage cellular proteins, RNA and DNA by forming adducts and cross-links, resulting in the loss of function of proteins and nucleic acids. The result is cytotoxicity (mainly the liver is damaged), mutagenicity and carcinogenicity. Sometimes, the lungs are also affected. The resulting formation of reactive oxygen species and the binding of pyrrolizidine alkaloids to the SH groups of proteins and enzymes probably further contribute to organ damage.

Clinical symptoms include fatigue, apathy, weight loss, and digestive problems including diarrhea, rectal prolapse, ascites, icterus, and photosensitive reactions on the skin and mucous membranes (secondary phototoxicity), and neurological symptoms may occur. These are characterized by a change in behavior (e.g., aggressiveness and pica), a change in posture, head pushing, ignoring obstacles while walking, etc. [5,11,27,28].

### 2.11. Plants Causing Allergic Reactions and Other Toxic Plants

#### 2.11.1. Allergenic Plants

**Figure 32:**
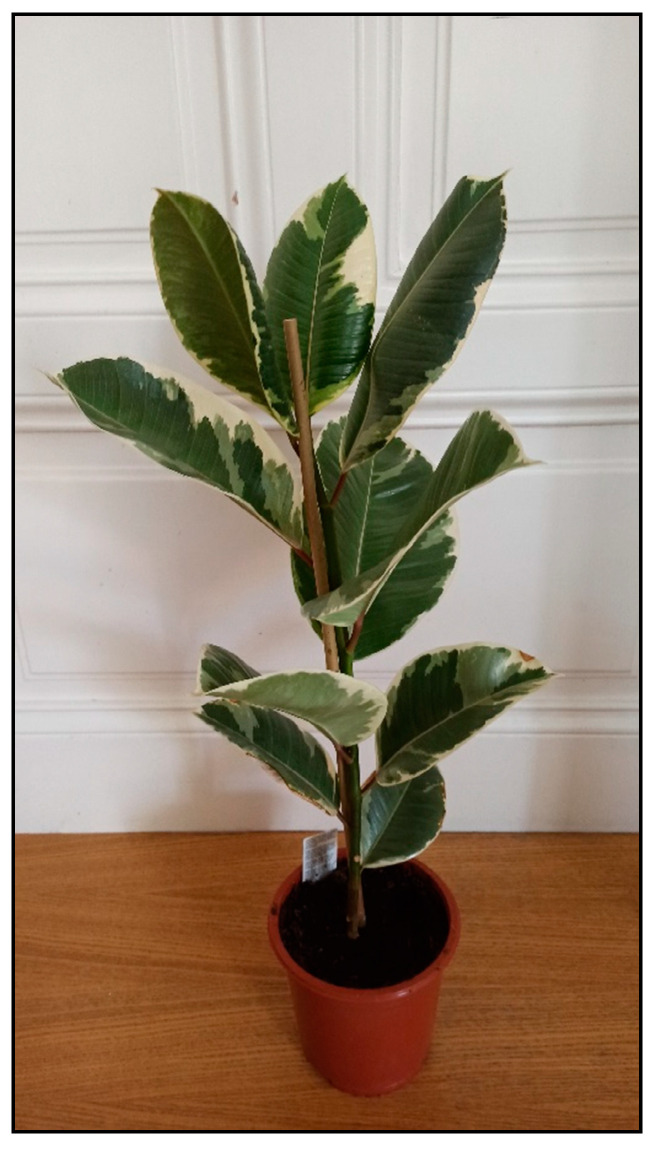
*Ficus elastica* Tineke (contains locally irritating substances; author: Zuzana Siroka).

**Figure 33:**
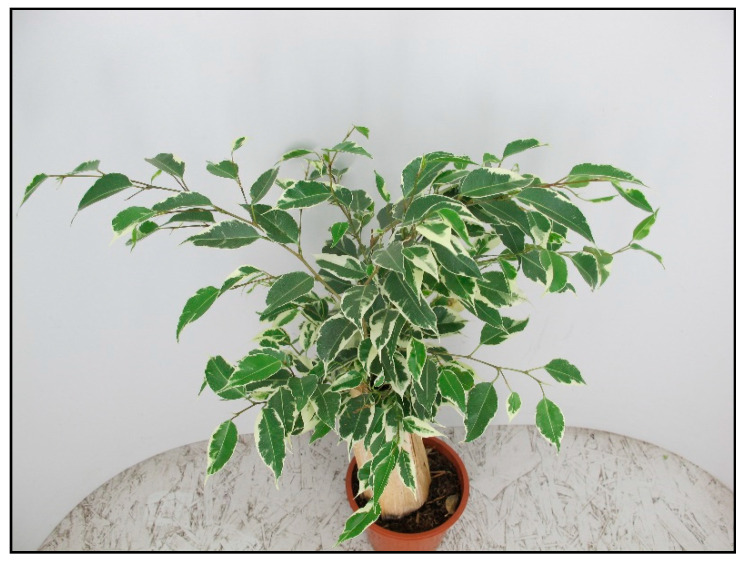
*Ficus benjamina* Kinky (contains locally irritating substances; author: Lenka Divisova).

Allergic reactions are usually caused by skin contact with the plant, but can also occur after ingesting the plant. The most common symptom is skin irritation. Representatives of this type of toxicity include some wax plants (*Hoya* spp.), chrysanthemums (*Chrysanthemum* spp.), coleus (*Coleus* spp.), ficuses/fig plants (*Ficus elastica—*Figure 32, *Ficus benjamina*—Figure 33, *Ficus benghalensis*, *Ficus lyrata*, etc.), poison primrose (*Primula obconica*), African milk bush (*Synadenium grantii*), aralia (*Polyscias* spp.), geranium (*Pelargonium* spp.), spiderwort and creeping inch plants (*Tradescantia* and *Callisia* spp.). or citrus plants [5,11].

#### 2.11.2. Other Toxic Plants That Are Grown in Households

**Figure 34:**
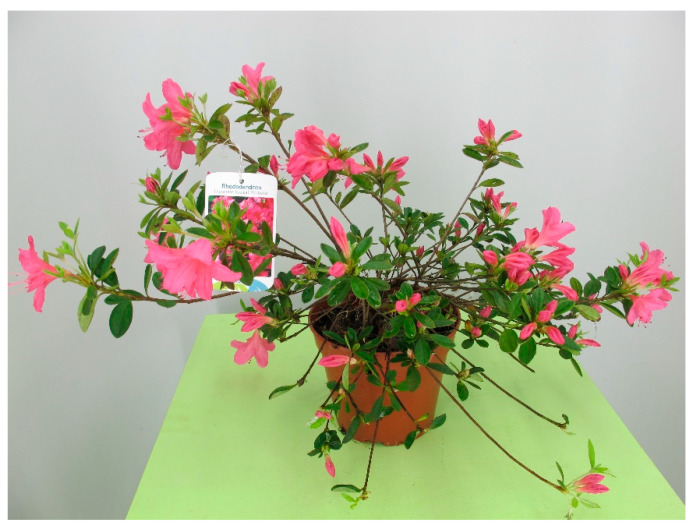
*Rhododendron* hybrid (contains diterpene grayanotoxin; author: Lenka Divisova).

Rhododendrons and azaleas (*Rhododendron* spp., Figure 34) contain diterpene grayanotoxin. Its mechanism of action is prolonged sodium channel activation and cell depolarization which then leads to overstimulation of the central nervous system. This causes salivation, vomiting, diarrhea, dyspnea, muscle weakness, convulsions, and comas. The cardiovascular effects caused by *nervus vagus* impairment may include hypotension, sinus bradycardia or bradyarrhythmia and partial or complete atrioventricular block. Signs may persist several days [11,15,29]. Some sources also mention miosis as a clinical sign [5].

**Figure 35:**
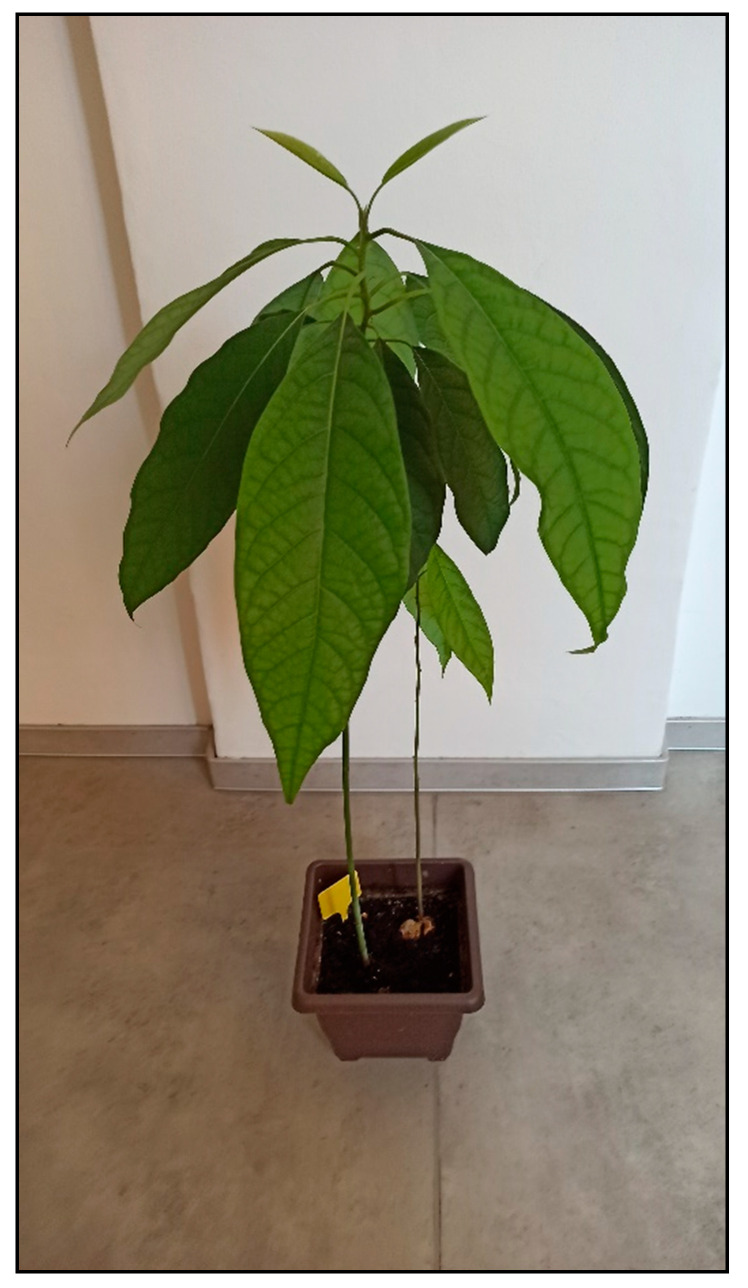
*Persea americana* (contains fatty acid derivate persine; author: Zuzana Siroka).

Avocado plant (*Persea americana*, Figure 35) is often grown at home in moderate to cold climate countries from the seed of the fruit bought for consumption. It contains the toxic fatty acid derivate persine with cardiotoxic effects which appear mainly in parrots or rabbits or small rodents. It causes unrest, weakness, pulmonary congestion or edema with dyspnea, cardiac arrhythmias, and often sudden death within 24 h after ingestion [15,30,31].

Glory lily/climbing lily (*Gloriosa superba*) is a climbing plant with antimitotic alkaloid colchicine in all its parts, which causes vomiting, diarrhea, dysphagia, oliguria, vasodilation and increased vascular permeability, thus inducing hypovolemic shock. Neurological signs such as convulsions, later paralysis and death due to respiratory or circulatory failure may occur. Several days after the onset of acute symptoms, alopecia and bone marrow depression with a decrease in blood cells may follow [5].

Aloe plants (*Aloe* spp.) are succulents containing gastrotoxic/laxative anthraquinones which cause vomiting, diarrhea, and sometimes also hypoglycemia [11,15].

## 3. Treatment

There is no specific antidote for most poisonous plants. Treatment is usually only symptomatic and supportive (including respiratory support, cardiovascular support or body temperature control).

As a first step, it includes the evacuation of stomach contents via inducing vomiting (in conscious animals, using hydrogen peroxide, apomorphine, etc.) or performing gastric lavage, especially if the animal does not vomit itself. These procedures are the most efficient within the first hour after plant ingestion. If more than 2 h has passed since ingestion, it is likely that the material has continued to the intestine and that the further inducement of vomiting will have no positive effect on the prognosis. Stomach content should always be reserved for examination, as the remains of the plant material in it may help with the identification of the ingested plant. Chemical analysis of the active substances both in digestive tract content or in the blood or urine of the affected animal is rarely performed, first due to its high price, and second due to the very limited availability of such analyses.

The second step is the administration of adsorbents (mainly activated charcoal at a dose of 1 g/kg of body weight), while rehydration and protective treatment of the digestive tract follows. Adsorbents may be administered repetitively in case the plant active substance undergoes an enterohepatic cycle (e.g., many cardiac glucosides). In case large doses of plant material are ingested, laxative agents (sodium sulfate, magnesium sulfate or sorbitol) can also be administered to enhance toxin elimination. However, care should be taken to avoid dehydration or electrolyte and acid-base imbalance in the patient. Fluid therapy can be supplied as a compensation for the fluid loss caused during intense vomiting or bowel evacuation.

In case of damage to the skin or mucous membranes, healing ointments and antihistamines are applied. If the eyes are affected, irrigation with a physiological solution is performed, and depending on the symptoms, antibiotics, local corticoids and artificial tears are added.

First aid for oxalate exposure should include rinsing or wiping the affected area with cold water. If the animal is swallowing and breathing normally, milk can be given to drink. Intensive monitoring of the upper respiratory tract’s condition is necessary. Depending on the severity of swelling in the throat, we administer corticoids by injection, or in life-threatening cases we perform intubation or a tracheotomy.

In cases of seizures caused by poisoning, anticonvulsants (diazepam, methocarbamol, barbiturates, and anesthetics—depending on the severity) must be given. In the case of hemolytic anemia (plants containing saponins), it is also possible to supplement the animal with vitamins of group B and folic acid, which serve as hepatoprotective agents and support for hematopoesis.

In cases of kidney failure (lily), it is necessary to introduce infusion therapy as quickly as possible, which should continue for at least 48 h. In cases of oliguria or anuria, it is recommended to perform hemodialysis or peritoneal dialysis. The prognosis is good in the case of early detection, but in the case of the development of kidney failure, it is uncertain to poor.

In the case of cardiotoxicity manifestations, it is necessary to check the electrolyte balance, monitor the heart functions, and administer atropine or antiarrhythmics according to electrocardiogram findings and the patient’s condition. Administration of calcium is contraindicated in the case of cardiac glycosides. Electrical cardioversion is usually ineffective.

In the case of the development of hepatoencephalopathy (e.g., with senecio plants), antioxidants, zinc, a diet containing proteins rich in sulfuric amino acids (but not a high-protein diet), silymarin and vitamins of group B are used [5,32].

## 4. Conclusions

Plant poisonings are often underdiagnosed and can be passed by unnoticed because of the non-specific clinical signs or the general unfamiliarity with toxic plants of both owners and veterinarians.

In the case of phone inquiries from owners, which are quite frequent in cases of plant ingestion/poisoning, it is necessary to inform the pet owner that if they cannot reliably identify the plant, the animal must always be taken to a veterinarian. Remote counselling and treatment are not possible, because it could be a highly toxic plant and only the direct intervention of a veterinarian can save the animal. It is always necessary to instruct the owner of the animal to bring the remaining part of the plant with them for identification, or at least to take a high-quality photo of the plant. If the plant is not identified, it makes it difficult to make a proper diagnosis, determine the prognosis and to apply more specific treatment [5]. Possible recent treatment of the plant with insecticides or fungicides must be also taken into consideration, and the veterinarian should ask the owner about such applications.

Furthermore, it is necessary to continuously educate veterinarians and the public so that they are able to identify poisonous plants, or at least the species of the plant. Scientific names of the genus/species should be preferred to common names of plants, which may be often misleading because they can be used for several different plant species. Then, it is necessary to appeal to the owners to place indoor plants containing toxic substances out of the reach of animals, and in case this is not possible and the animal is interested in plants (not only nibbling them, but also rubbing them, playing with them, etc.), it is necessary to remove toxic plants from the household. Attention should also be paid to seasonal decorative plants such as mistletoe, Christmas star, etc., which may be new to the household and unknown for the animal; moreover, they are often placed on the table, so are easily accessible to the animal, which may be overly interested in them. Prevention is the best way to avoid these poisonings and should play an essential role.

Another appropriate step would be mandatory labeling of the correct species of plant (names are often confused, or there is no identification on the package) and the toxicity of the plant at the sellers’, which is still very rare, because it is not required by applicable legislation. People would then not only buy plants based on their appearance, but could also choose them based on their suitability for placement in a family with animals and small children.

## Data Availability

Not applicable.
